# Association of Obesity Indicators with Hypertension in Type 2 Diabetes Mellitus Patients

**DOI:** 10.7759/cureus.5050

**Published:** 2019-07-01

**Authors:** Ghulam Mohyud Din Chaudhary, Asim Tameez Ud Din, Farooq Mohyud Din Chaudhary, Azfar Tanveer, Khaleeq H Siddiqui, Asma Tameez Ud Din, Noman A Chaudhary, Sana Mohyud Din Chaudhary, Ahsan Tameez-ud-din, Faisal Nawaz

**Affiliations:** 1 Internal Medicine, Nishtar Medical University & Hospital, Multan, PAK; 2 Internal Medicine, Rawalpindi Medical University, Rawalpindi, PAK; 3 Gastroenterology, Nishtar Medical University & Hospital, Multan, PAK; 4 Internal Medicine, NewYork-Presbyterian Queens, Flushing, USA; 5 Surgery, Rawalpindi Medical University, Rawalpindi, PAK; 6 Internal Medicine, Combined Military Hospital Lahore Medical College & Institute of Dentistry, Lahore, PAK; 7 Gastroenterology, Good Hope Hospital, University Hospitals Birmingham, Birmingham, GBR

**Keywords:** type 2 diabetes mellitus, hypertension, obesity, waist circumference, waist to hip ratio, body mass index

## Abstract

Objectives: To study the association of waist circumference (WC), waist to hip ratio (WHR) and body mass index (BMI) with hypertension in type 2 diabetes mellitus (DM) patients in a tertiary care hospital.

Methods: The anthropometric measures of patients were recorded in the Diabetic Outdoor of Nishtar Hospital Multan from 2013 to 2018 after taking approval from the Institutional Ethical Review Committee. All patients were evaluated in detail after obtaining informed consent. Data was entered and analyzed in SPSS version 20 (IBM Corp., Armonk, NY, USA).

Results: Data of 4556 type 2 DM patients, 2549 (55.9%) females, and 2007 (44.1%) males, was analyzed. Mean age of the study population was 47.72 years. Mean age of females was 47.32 years, while of males was 48.23 years. A total of 3393 (74.5%) of the patients had hypertension, 1912 females and 1481 males. The mean systolic blood pressure (SBP) was 130.84 mmHg, while the mean diastolic blood pressure (DBP) was 82.65 mmHg. Mean WC was 102.85 cm. Mean hip circumference was 100.33 cm. Mean weight was 66.93 kg. Mean height was 1.59 m. Mean WHR was 1.02. Mean BMI was 26.37 kg/m2. Obesity (BMI >27 kg/m2) was found in 1,891 (41.5%) of patients. Central obesity was found in 80.7% and 94.7% of type 2 DM patients according to the WC and WHR cutoff, respectively. Hypertension was significantly associated with all the obesity indicators (p<0.001). Type 2 DM patients with a high WHR were more likely to be hypertensive as compared to those with normal WHR (75% versus 65%, odds ratio (OR) 1.6, p<0.001). A higher than normal WC was also significantly associated with hypertension (79% versus 56%, OR 2.9, p<0.001). Similarly, obese type 2 DM patients with a BMI >27 kg/m2 were more likely to be hypertensive as compared to those with a normal range (18.5 to 22.9 kg/m2) BMI (83.1% versus 64.4%, OR 2.7, p<0.001).

Conclusion: Diabetes is more prevalent in females and middle-aged people. Hypertension and obesity are two very common comorbidities of diabetes. Hypertension is strongly associated with all the parameters (WC, WHR, and BMI) of obesity.

## Introduction

Diabetes mellitus (DM) has emerged as one of the global health issues in recent years. Its prevalence in adults is reported to be 8% which is expected to rise to 10% by 2040. More than 90% of this population belongs to type 2 DM group [1]. The pathophysiology of Type 2 DM mainly involves insulin resistance. It is associated with multiple cardiovascular and metabolic risk factors [2]. Obesity has emerged as one of the strongly associated modifiable risk factors of type 2 DM owing to its role in insulin resistance. By 2025, there are reports that obesity, which is related to diabetes, would rise to an alarming number of 300 million [3].

Hypertension is another risk factor associated with type 2 DM. Its prevalence in diabetic patients is almost double as compared to non-diabetics [4]. The presence of hypertension expedites the development of complications in a diabetic patient like stroke, myocardial infarction, retinopathy, neuropathy, and nephropathy. The concomitant presence of hypertension in a diabetic patient increases the mortality and morbidity of these patients [5].

There are multiple studies evaluating obesity and hypertension as independent risk factors to DM. But the association of obesity and hypertension in a diabetic patient is not widely studied. In this article, we aim to demonstrate the association of different indicators of obesity like waist circumference (WC), waist to hip ratio (WHR), and body mass index (BMI) with hypertension in type 2 DM patients.

## Materials and methods

This descriptive analytical study was carried out at the Diabetic Outdoor Nishtar Hospital Multan after taking approval from the local Institutional Ethical Committee. After obtaining informed consent, data of 4556 type 2 DM patients presenting to the Diabetes Clinic was collected from 2013 to 2018. The inclusion criteria included all adults of age 18 years or older, who were either previously diagnosed diabetics or newly diagnosed type 2 DM. Diabetes was diagnosed according to the following cut-off values; fasting blood sugar (FBS) >126 mg/dl and random blood sugar (RBS) >200 mg/dL. Patients less than 18 years of age, or those who had type 1 DM, impaired glucose tolerance, impaired fasting glucose, or gestational DM were excluded from the study. Patient's history, examination findings, and laboratory investigations were evaluated in meticulous detail.

A predesigned performa was used to record the demographic and anthropometric measures of the patients. Hypertension was defined according to American College of Cardiology/American Heart Association (ACC/AHA) guidelines: systolic blood pressure (SBP) >130 mmHg or diastolic blood pressure (DBP) >80 mmHg [6]. Body mass index (BMI) was calculated by the formula: weight(kg)/height(m2). Obesity was defined as BMI >27 kg/m2 according to BMI cutoff for Asians [7]. Central obesity was defined according to the following cut-off values: waist circumference (WC; men ≥90 cm and women ≥80 cm) and waist to hip ratio (WHR; men > 0.9, women > 0.8) [8-9]. The gathered data was entered in SPSS version 20 (IBM Corp., Armonk, NY, USA). Statistical data analysis was performed with chi-square. Statistical significance was determined at p <0.05. Information obtained was then analyzed. Association of hypertension with different demographic variables and obesity parameters was evaluated by calculating the odds ratio (OR). The findings were presented in the form of tables.

## Results

Out of 4556 patients with Type 2 DM, 2549 (55.9%) were females while 2007 (44.1%) were males. Mean age of the patients was 47.72±10.82 years with a range of 18-95 years. Mean age of females was 47.32±10.37 years, while that of males was 48.23±11.35 years.

A total of 3393 (74.5%) of the patients had hypertension. Out of 3393 patients, 1912 (56.35%) were females while 1481 (43.65%) were males. The mean SBP was 130.84±29.64 mmHg, while the mean DBP was 82.65±19.36 mmHg. Means and standard deviation of indicators of obesity are shown in Table 1.

**Table 1:** Means and standard deviation of indicators of obesity cm: centimeters; kg: kilograms; m: meters; kg/m2: kilograms per meter square.

Indicators of Obesity	Mean	Standard Deviation
Waist circumference (WC)	102.85 cm	18.14 cm
Hip circumference	100.33 cm	11.81 cm
Weight	66.93 kg	14.92 kg
Height	1.59 m	0.159 m
Waist to hip ratio (WHR)	1.02	0.102
Body mass index (BMI)	26.37 kg/m^2^	5.86 kg/m^2^

Gender of the patient was not associated significantly with hypertension (p=0.35). However, hypertension was found to be strongly associated with increasing age. Type 2 DM patients belonging to middle and older age groups were more likely to hypertensive as compared to the younger age group (74-76% versus 41.7%, OR 4.5, p< 0.001). Central obesity was found in 80.7% and 94.7% of type 2 DM patients according to the WC and WHR cutoff, respectively. Hypertension was significantly associated with all the obesity indicators (p<0.001). Type 2 DM patients with a high WHR were more likely to be hypertensive as compared to those with normal WHR (75% versus 65%, odds ratio (OR) 1.6, p<0.001). A higher than normal WC was also significantly associated with hypertension (79% versus 56%, OR 2.9, p<0.001). Similarly, obese type 2 DM patients with a BMI >27 kg/m2 were more likely to be hypertensive as compared to those with a BMI of less than 18.5 (83.1% versus 55.9%, OR 3.9, p<0.001). Table 2 shows the association of age, gender and different parameters of obesity with hypertension in patients with type 2 DM.

**Table 2:** Association of age, gender, and different parameters of obesity with hypertension in patients with type 2 diabetes mellitus (DM) WC: waist circumference; WHR: waist to hip ratio; BMI: body mass index; cm: centimeters.

		Blood Pressure			
Variable		Normotensive N(%)	Hypertensive N(%)	Odds ratio	P value
Age (years)	18-20	21 (58.3%)	15 (41.7%)	1	<0.001
	21-30	93 (36.8%)	160 (63.2%)	2.4	
	31-40	268 (25.9%)	765 (74.1%)	4	
	41-50	426 (24.0%)	1346 (76.0%)	4.4	
	51-60	256 (23.7%)	822 (76.3%)	4.5	
	>60	99 (25.8%)	285 (74.2%)	4	
Gender	Female	637 (25.0%)	1912 (75.0%)	1	0.35
	Male	526 (26.2%)	1481 (73.8%)	0.9	
WC (cm)	Normal	383 (43.6%)	496 (56.4%)	1	<0.001
	High	780 (21.2%)	2897 (78.8%)	2.9	
WHR	Normal	86 (35.4%)	157 (64.6%)	1	<0.001
	High	1077 (25.0%)	3236 (75.0%)	1.6	
BMI (kg/m^2^)	<18.5	113 (44.1%)	143 (55.9%)	1	<0.001
	18.5-22.99	339 (35.6%)	614 (64.4%)	1.4	
	23-26.99	392 (26.9%)	1064 (73.1%)	2.1	
	≥27	319 (16.9%)	1572 (83.1%)	3.9	

## Discussion

In our study, the mean age of type 2 diabetic patients was 47.72±10.82 years which is lower than similar studies done in the West and Middle East [10-12]. This may be attributed to dietary habits, genetics, and other environmental factors. Our study indicates a high prevalence of hypertension in females (75%) as compared to males (73.8%) but it was not statistically significant. This result is different from other studies. In a study conducted in Brazil, hypertension was found to be more prevalent in males (34%) as compared to females (30.8%) [13]. Similar results of high prevalence of hypertension in males were found in studies conducted in Argentina, China, and England [14-16]. The difference could be because of multiple factors involving presence or absence of diabetes, socioeconomic status, differences in educational status and environmental factors. This needs to be further investigated. Another important finding in our study was a significant association of hypertension with age. In our study, as soon as the patient enters the middle age group and beyond that, the prevalence of hypertension ranges from 74% to 76%. No statistically significant difference in prevalence is found in the middle age and old age group of diabetics in our study. However, when compared to young age, the difference becomes significant (74% versus 41%, OR 4, p<0.001). This is in contrast with the data obtained from National Health and Nutrition Examination Survey where 70% of the older patients have hypertension in contrast to 32% of the patients whose ages were between 40-59 years [17].

Our study indicated a statistically significant association of hypertension with all the obesity indicators. In our study, 83.1% of the patients who had high BMI (≥ 27) were found to be hypertensive. Similar results were found in a cross-sectional study conducted on type 2 diabetic patients. In that study where obesity was defined as BMI ≥ 30 kg/m2, 86.2% were found to be hypertensive [10]. Another obesity indicator used in our study was WC. The WC of ≥90 cm in men and ≥80 cm in women were considered to be obese. In our study, 78.8% of patients with high WC were hypertensive as compared to 56.4% with normal WC. This was comparable to another study in which abdominal obesity was defined as WC of ≥ 102 cm in men and ≥ 88 cm in women. That study found 81.5% of obese patients to be hypertensive as compared to 63.3% non-obese [10]. Similar results were also found in studies conducted in Sweden and Jordan [11-12]. Another indicator defined in our study was WHR. Seventy-five percent of patients with high WHR were found to be hypertensive as compared to 64.6% with normal WHR. There is not much data found regarding the use of WHR as an obesity indicator in relation to hypertension in type 2 diabetic patients. But there are multiple studies showing a strong association of WHR with hypertension in general population [18-19].

This study had some limitations. It was carried out at a tertiary care center so it may not represent the general population. It was conducted in a single center (diabetic outdoor of Nishtar Hospital Multan, Pakistan). Multi-center studies should be done to further explore this topic.

## Conclusions

Diabetes affects people from all walks of life. It is a global health issue and has become a challenge especially in developing countries like Pakistan. Our study showed that DM is more prevalent in females and middle age groups. There was an alarmingly high prevalence of hypertension and obesity in type 2 DM patients in our study. Again, females were more likely to have hypertension as compared to male diabetics. Central obesity was a more worrisome problem as it affected more than 80% of diabetics in our study. A very strong association was observed between obesity and hypertension in type 2 DM patients. Hypertension was significantly associated (p<0.001) with all the parameters of obesity (WC, WHR, and BMI). Hypertension was also found to be more prevalent in middle and old age diabetics as compared to young type 2 DM patients. Awareness and education in society are of immense importance if we are to control the growing problems of obesity and hypertension in type 2 diabetes patients.
